# Toxicology of diatomaceous earth, phyto oils and their admixed emulsions against adults of *Tribolium castaneum* (Herbst)

**DOI:** 10.1016/j.toxrep.2022.05.011

**Published:** 2022-05-16

**Authors:** Maryam Tanveer, Shaghef Ejaz, Syed Muhammad Zaka, Muazzama Batool, Tatheer e Zahra, Muhammad Saghir, Qamar Saeed

**Affiliations:** a Department of Entomology, Faculty of Agricultural Sciences & Technology, Bahauddin Zakariya University, 60800 Multan, Pakistan; b Department of Horticulture, Faculty of Agricultural Sciences & Technology, Bahauddin Zakariya University, 60800 Multan, Pakistan

**Keywords:** Essential oil, Efficacy, Emulsion, Toxicity, *T. castaneum*

## Abstract

*Tribolium castaneum* (Herbst), one of the invasive stored pest, is resistant to the most of synthetic insecticides used against this it. Moreover, synthetic insecticides are a major threat to human health, the environment, and the ecosystem. The development of some smart tools is needed to minimize the use of hazardous chemicals. As an alternative, nano-insecticides are on the horizon. Emulsions are expressed as sustain release of insecticidal components to achieve maximum efficacy and low residual toxicity. In this study, some essential oils (*Cymbopogon citratus* (DC.) Stapf, *Ocimum basilicum* L., *Curcuma longa* L., and *Trachyspermum ammi* L.), diatomaceous earth (DE), and their nano-emulsions are evaluated against *T. castaneum*. Essential oils and DE were tested at four (60 ppm, 30 ppm, 15 ppm, 7.5 ppm) different concentrations with and without emulsions, and data was recorded after 6, 12, 24, 48, and 72 h of exposure respectively. The mortality observed in essential oils of *C. citratus*, *O. basilicum*, and *C. longa* without emulsion after 72 h of treatment at the highest concentration was 98%, 95%, and 85%, respectively. While, at the highest concentration the lowest mortalities were observed with DE and *T. ammi* i.e. 65%. Insecticidal activity of emulsion essential oils increased to 100%, 98%, 90%, and 68.3% for *C. longa*, *C. citratus*, *O. basilicum*, and *T. ammi*, respectively. The results support that these admixed emulsions could be used as an alternative to synthetic insecticides in conventional formulations.

## Highlights

• Plant oils could be an alternate to synthetic insecticides against stored grain insect pests.
• Limitations to botanical pesticides can be overcome through new formulations.
• Development of plant oils as nano-emulsions could significantly enhance their penetration at target site.

## Graphical Abstract

**Figure:**
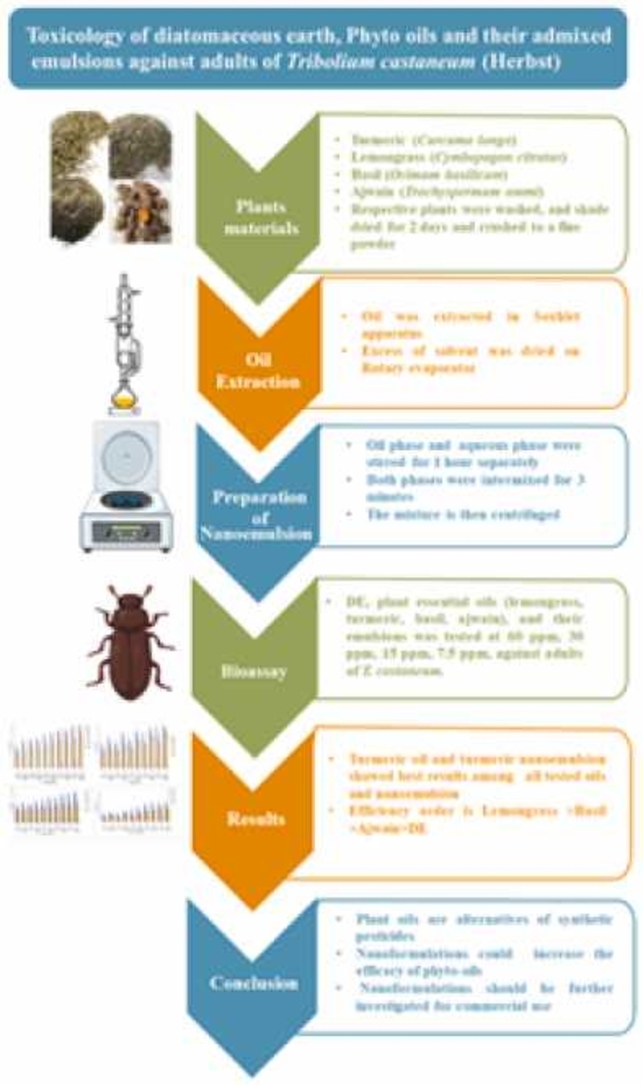

## 1 Introduction

Food production must be increased to feed the ever-increasing population. Post-harvest losses by insect pests account for 25–50% [1], [2]. *Tribolium castaneum* (Herbst) is one of the most common pest of stored grains and is known to infests about 233 different types of crops [3]. The *T. castaneum* is responsible for considerable losses in stored wheat [4], [5], [6]. The most applied method for its control is fumigation [7]. Among fumigants, methyl bromide is discontinued due to ozone depletion effects [8]. The *T. castaneum* has developed resistance against malathion, cypermethrin, primiphos-methyl, and bifenthrin [9], [10]. Alternate to the pesticides are biopesticides, which can be used safely against the insect pests to reduce the limitations of synthetic pesticides [11].

Plants produce substances for their defense against pests which upon extraction can be used as biopesticides [12], [13], [14], [15]. The *C. citratus* (Lemongrass) is a commonly found plant with variety of medicinal uses [16], [17]. The essential oils derived from lemongrass are also reported to possess insecticidal activity due to the presence of E-citral [18], [19]. The *C. longa* (Turmeric) is known for uses in cosmetics, medicine and pesticide industry [20], [21], [22], [23], [24], [25], [26], [27]. *Ar*-turmerone is the most effective component responsible for insecticidal activity [28], [29]. Essential oils extracted from *O. basilicum* (basil) and *T. ammi* (ajwain) are reported for having repellents and antifeedants actions against different types pests [30], [31], [32], [33], [34], [35], [36]. Linalool in basil and thymol in ajwain is also responsible for its insecticidal activity [31], [32], [33], [37], [38]. Plant-extracted essential oils act synergistically with insecticides while having increased shelf life [39], [40].

Diatoms are the most abundant algae [41]. They are advantageous over chemical pesticides being non-hazardous to non-target organisms [42]. The DE (diatomaceous earth) absorbs all liquid content in insect cuticles and leads them to death [43], [44], [45], [46]. Nano and microencapsulation technologies are employed widely to increase the availability and stability of natural substances in essential oils [47]. The nano formulated biopesticides have a controlled release mechanism to exhibit maximum efficacy [48]. Nanofabrication of insecticidal substances can increase the insecticidal effect by improving the delivery of active ingredients to the target site because of ultra-small particles which can even pass through extracellular spaces [49].

The study was designed to estimate the insecticidal efficacy of the above-stated oils and DE against *T. castaneum*. The present study consequently focused on the development of new formulations by using technology to improve the efficacy of these biopesticides which could be an important contribution to agriculture.

## 2 Materials and methods

### 2.1 Insect culture

Infested wheat flour was collected from the local market of Multan. Adults of *T. castaneum* were sieved and transferred to rearing jars supplied with a mixture of flour (sterilized) and 5% yeast in Ecotoxicology Laboratory, Department of Entomology, Bahauddin Zakariya University, Multan. The rearing jars were kept at 28 ± 2 ºC and 70% R.H. for insect multiplication [50]. Pupae were separated and kept in batches to get homogenous population. One week old adults of *T. castaneum* were collected through pit fall traps and starved for 24 h before treatment.

### 2.2 Plant materials

Leaves of *C. citratus* (lemongrass), rhizomes of *C. longa* (turmeric), seeds of *O. basilicum* (basil), and *T. ammi* (ajwain) were purchased from the local market of Cantt, Multan, Pakistan. Leaves, rhizomes and seeds of respective plants were washed, and shade dried for 2 days and crushed by a grinding mill to a fine powder. (Table 1).

**Table 1:** List of plant species and their major insecticidal compound used against *T. castaneum*.

**Common names**	**Scientific names**	**Families**	**Major insecticidal components**	**References**
Lemongrass	*Cymbopogon citratus*	Poaceae	E-citral	[18]
Turmeric	*Curcuma longa*	Zingiberaceae	*Ar*-Tumerone	[28], [29]
Basil	*Ocimum basilicum*	Lamiaceae	Linalool	[37]
Ajwain	*Trachyspermum ammi*	Apiaceae	Thymol	[38]

### 2.3 Oil extraction

Dried lemongrass leaves powder, turmeric rhizomes, ajwain and basil seed powders (20 g) were suspended in 350 ml acetone at 56 ºC for 12, 6, 8 and 8 h respectively, in the Soxhlet apparatus. Excess solvent was removed by placing the mixture (in a flask) over the rotary evaporator at 56 ºC and 25 rpm to dry excess solvent The concentrate obtained was stored in glass vials at 4 ºC [51], [52], [53].

### 2.4 Preparation of admixed emulsions

The oils extracted were then utilized to make admixed emulsions. They were made in two phases because being inorganic DE could not be mixed directly with test oils. The oil phase constituted 14% oil, 3% ethanol, and 3% Tween 20 representing 20% of the emulsion. The oil phase was stirred at 86 ºC and 750 rpm of 1 h. While the aqueous phase was prepared by mixing of 2 g of DE in 78 ml of water and was stirred at 86 ºC and 75 rpm for 1 h. Both aqueous and oil phases were intermixed at the same temperature for 3 min. The emulsion thus prepared was centrifuged at 10,000 *xg* for 30 min and the emulsion was stored in dark bottles [54], [55].

### 2.5 Bioassays

The efficiency of DE, plant essential oils (lemongrass, turmeric, basil, ajwain), and their emulsions was tested at four serially diluted concentration i.e., 60 ppm, 30 ppm, 15 ppm and 7.5 ppm against adults of *T. castaneum*. Control was treated with distilled water designated as 0 ppm. The experiment was performed using a completely randomized design with 6 replications of each treatment. Filter papers of 5 cm diameter were treated and air-dried for 1 h. Dried filter papers were kept in already sanitized same diameter petri dishes and 10 insects were placed in each petri dish. The petri dishes were kept at room temperature and the mortality data was taken at intervals of 6, 12, 24, 48, and 72 h, respectively [56].

### 2.6 Statistical analysis

Data were analyzed through Two-Way ANOVA in Minitab 19 Software. Mean mortalities were compared by LSD to find homogenous groups [57]. The toxicity of different treatments (LC_50_) was determined by Probit analysis.

## 3 Results

### 3.1 Efficacy of lemongrass oil and emulsion

**Fig. 1:**
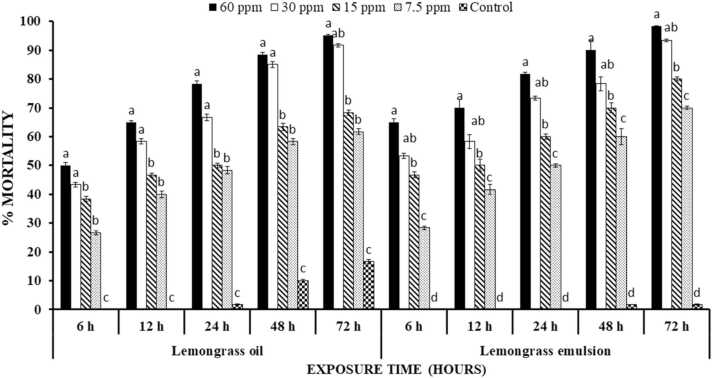
Percentage mortality ( ± S.E.) of *T. castaneum* treated with lemongrass oil and emulsion at different time intervals. Letterings on each time interval is representing one analysis and bars having different lettering representing significance difference (P < 0.00001) among different concentrations.

Adults of *T. castaneum,* treated with different concentrations of lemongrass oil, showed significant results at different intervals of time. Significantly (P < 0.0001) highest mortalities were observed at 60 ppm i.e., 50%, 60%, 70% and 80% at 6, 12, 24 and 48 h, respectively. Similarly, subsequent lower concentrations of lemongrass oil showed lower mortalities (Fig. 1). Different treatments of lemongrass emulsion exhibited relatively higher mortalities (P < 0.0001) as compared to lemongrass oil treatments (Fig. 1).

### 3.2 Efficacy of turmeric oil and emulsion

Percentage mortality of *T. castaneum* after treatment with turmeric essential oil significantly increased with increase in concentration (P < 0.00001) and time Percentage mortality of *T. castaneum* observed after exposure to turmeric oil was 56%, 63.3%, 70%, 83.3% and 98.3% at 6, 12, 24, 48 and 72 h of treatment at highest concentration.

**Fig. 2:**
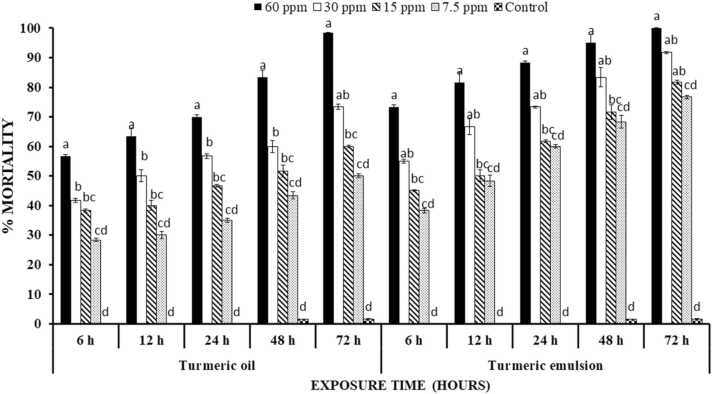
Percentage mortality ( ± S.E.) of *T. castaneum* treated with turmeric oil and emulsion at different time intervals. Letterings on each time interval is representing one analysis and bars having different lettering representing significance difference (P < 0.00001) among different concentrations.

The emulsions of turmeric oil expressed comparatively high mortality (P < 0.0001) than turmeric oil, while the pattern of mortality percentage was dependent on time lapse after treatment and concentration. At 60 ppm percentage mortality of turmeric emulsion was increased at 17.33% i.e., 73.33% after 6 h of treatment as compared to 56% mortality of turmeric oil at same concentration and time interval. Other concentrations and time intervals expressed subsequent pattern of observation statistically significant from each other and control (P < 0.0001) (Fig. 2).

### 3.3 Efficacy of basil oil and emulsion

**Fig. 3:**
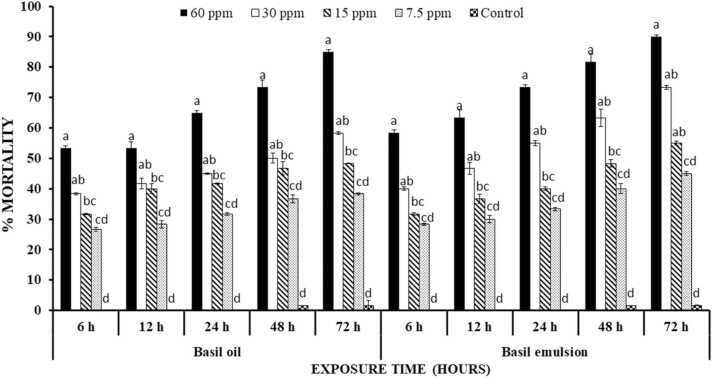
Percentage mortality ( ± S.E.) of *T. castaneum* treated with basil oil and emulsion at different time intervals. Letterings on each time interval is representing one analysis and bars having different lettering representing significance difference (P < 0.00001) among different concentrations.

Basil essential oil was found significantly effective (P < 0.00001) against *T. castaneum* at tested concentrations (60, 30, 15 and 7.5 ppm) and time interval. The percentage mortality increased with increase in concentration and time after treated with basil oil. Basil emulsions treatments were recorded to have higher percentage mortality than basil emulsion treated units. Basil oil expressed 53.3% mortality after 6 h of treatment at 60 ppm while emulsion exhibited 58.3% at same conditions. Other concentrations of basil oil and emulsion showed similar pattern of mortality. All the treatments were statistically different from each other and control (P < 0.00001) (Fig. 3).

### 3.4 Efficacy of ajwain oil and emulsion

**Fig. 4:**
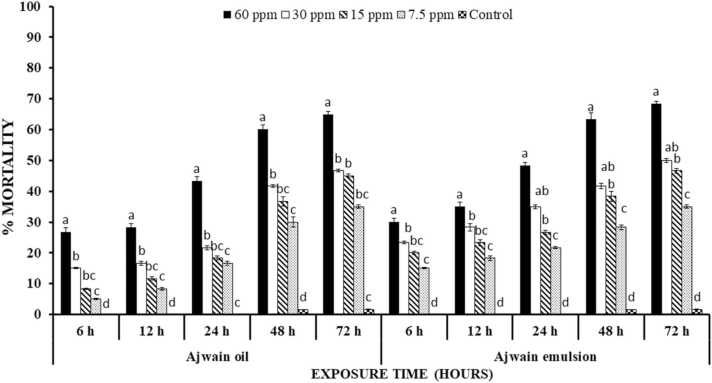
Percentage mortality ( ± S.E.) of *T. castaneum* treated with ajwain oil and emulsion at different time intervals. Letterings on each time interval is representing one analysis and bars having different lettering representing significance difference (P < 0.0001) among different concentrations.

Percentage mortality of *T. castaneum* after treatment with ajwain oil and emulsion significantly increased with increase in concentration and time (P < 0.00001). Emulsion of ajwain oil resulted 3.4% increased mortality i.e., 30% as compared to ajwain oil (26.67%) at highest concentration after 6 h of treatment. All treatments exhibited similar trend of percentage mortality while being significantly different from each other (P < 0.00001) (Fig. 4).

### 3.5 Efficacy of diatomaceous earth

**Fig. 5:**
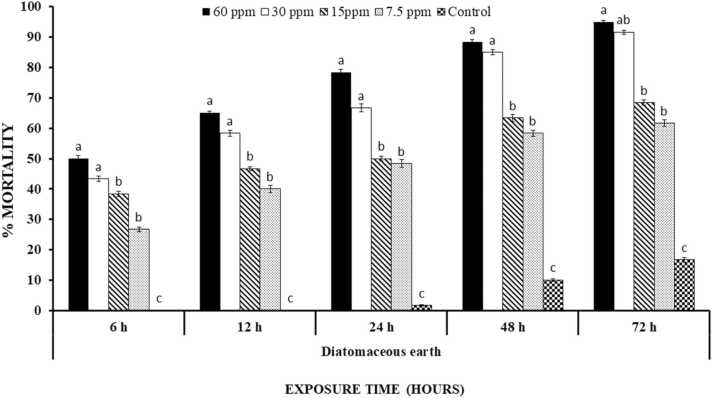
Percentage mortality ( ± S.E.) of *T. castaneum* treated with diatomaceous earth at different time intervals. Letterings on each time interval is representing one analysis and bars having different lettering representing significance difference (P < 0.00001) among different concentrations.

Percentage mortality of *T. castaneum* after treatment with diatomaceous earth significantly increased with an increase in concentration and time (P < 0.0001). The 60 ppm concentration of DE resulted in 20% mortality at 6 h interval. Percentage mortality was recorded as 23.33%, 56.67%, and 65% in DE at 60 ppm concentration after 24, 48 and 72 h of treatment, respectively. All the tested concentrations expressed parallel trend of mortality. Percentage mortality of *T. castaneum* in control was 1.67% after 72 h of treatment (Fig. 5).

### 3.6 Toxicity of plant-based oils and their emulsions against *T. castaneum*

The results of the study indicated that LC_50_ of *T. castaneum* was dependent on post treatment exposure time (Table 2, Table 3). Among oil treatments, turmeric oil was most toxic against *T. castaneum* with the lowest LC_50_ values ranging from 37.036 ppm, 20.650 ppm, 18.633 ppm, 13.683 ppm and 10.107 ppm at 6,12,24,48 and 72 h intervals respectively. The toxicity descended by lemongrass oil ˃ basil oil ˃ ajwain oil. The toxicity of DE was less then all tested oils ranging 614.842–72.074 ppm. The most toxic among all emulsions tested was turmeric emulsion. Turmeric emulsion has lowest LC_50_ values i.e. 17.593 ppm, 10.684 ppm, 5.761 ppm, 4.200 ppm and 1.787 ppm at 6,12,24,48 and 72 h interval. The descending order of emulsions toxicity was observed as lemongrass emulsion ˃ basil emulsion ˃ ajwain emulsion. The lowest toxicity recorded with ajwain emulsion was 71.747 ppm, 47.973 ppm, 29.602 ppm, 17.117 ppm and 14.613 ppm at 6,12,24,48 and 72 h respectively.

**Table 2:** Analysis of insecticidal effect of plant oils and diatomaceous earth with their respective lethal concentrations, fiducial limits (95%), chi-square (χ^2^) and slope ( ± standard error) after an exposure of 6, 12, 24, 48 and 72 h.

**Treatment**	**Exposure time (hours)**	**LC**_**50**_**(95% FL) (ppm)**	**df**^a^	**Slope ± SE**	**χ**^**2**^^b^
Lemongrass oil	6	42.04 (12.77–138.32)	2	0.76 ± 0.26	0.924
	12	27.67 (10.54–72.60)	2	0.94 ± 0.21	0.992
	24	18.63 (7.41–46.84)	2	0.99 ± 0.20	0.994
	48	6.05 (2.59–14.11)	2	1.16 ± 0.18	0.923
	72	5.47 (2.88–3.66)	2	1.73 ± 0.14	0.984
Turmeric oil	6	37.03 (11.90–115.22)	2	0.80 ± 0.25	0.873
	12	20.65 (8.20–51.98)	2	0.98 ± 0.20	0.994
	24	18.63 (7.41–46.84)	2	0.99 ± 0.20	0.994
	48	13.68 (6.32–30.05)	2	1.17 ± 0.17	0.838
	72	10.10 (6.04–16.90)	2	1.99 ± 0.11	0.644
Basil oil	6	57.02 (17.05–190.68)	2	0.76 ± 0.26	0.907
	12	48.14 (12.31–188.24)	2	0.69 ± 0.30	0.908
	24	28.02 (10.04–78.18)	2	0.88 ± 0.22	0.868
	48	21.02 (8.31–53.17)	2	0.98 ± 0.20	0.824
	72	15.846 (8.07–31.08)	2	1.38 ± 0.14	0.786
Ajwain oil	6	229.34 (82.45–637.93)	2	1.13 ± 0.22	0.955
	12	140.73 (53.82–367.99)	2	1.09 ± 0.21	0.942
	24	87.40 (35.08–217.77)	2	1.08 ± 0.20	0.930
	48	41.86 (17.36–100.90)	2	1.04 ± 0.19	0.880
	72	25.76 (10.70–62.03)	2	1.04 ± 0.19	0.906
Diatomaceous earth	6	614.84 (151.93–2488.06)	2	0.86 ± 0.31	0.961
	12	317.23 (92.28–1090.45)	2	0.89 ± 0.27	0.928
	24	261.34 (72.80–938.17)	2	0.81 ± 0.28	0.974
	48	127.13 (40.16–402.42)	2	0.85 ± 0.25	0.982
	72	72.07(23.64–219.65)	2	0.84 ± 0.24	0.945

a Degree of freedom

b Chi square

**Table 3:** Analysis of insecticidal effect of plant oil admixed emulsions with their respective lethal concentrations, fiducial limits (95%), chi-square (χ^2^) and slope ( ± standard error) after an exposure of 6, 12, 24, 48 and 72 h.

**Treatment**	**Exposure time (hours)**	**LC**_**50**_**(95% FL) (ppm)**	**df**^a^	**Slope ± SE**	**χ**^**2**^^b^
Lemongrass emulsion	6	23.58 (9.55–58.22)	2	1.01 ± 0.20	0.880
	12	18.45 (7.82–43.50)	2	1.06 ± 0.19	0.923
	24	11.09 (4.88–25.20)	2	1.13 ± 0.18	0.919
	48	6.89 (3.05–15.58)	2	1.19 ± 0.18	0.972
	72	4.87 (2.44–9.73)	2	1.59 ± 0.15	0.992
Turmeric emulsion	6	17.59 (7.05–43.87)	2	1.00 ± 0.20	0.899
	12	10.68 (4.52–25.23)	2	1.08 ± 0.19	0.819
	24	5.76 (2.27–143.59)	2	1.04 ± 0.20	0.883
	48	4.20 (1.76–10.00)	2	1.19 ± 0.19	0.933
	72	1.78 (0.58–5.42)	2	1.00 ± 0.24	0.885
Basil emulsion	6	42.90 (17.88–102.93)	2	1.06 ± 0.19	0.939
	12	31.58 (13.62–73.18)	2	1.09 ± 0.18	0.977
	24	21.49 (10.38–44.51)	2	1.26 ± 0.16	0.939
	48	15.94 (7.99–31.82)	2	1.34 ± 0.15	0.948
	72	11.21 (5.94–21.14)	2	1.51 ± 0.14	0.892
Ajwain emulsion	6	71.74 (32.03–160.71)	2	1.22 ± 0.17	0.996
	12	47.97 (20.84–110.42)	2	1.13 ± 0.18	0.995
	24	29.60 (11.85–73.94)	2	1.01 ± 0.20	0.981
	48	17.11 (7.36–39.77)	2	1.08 ± 0.18	0.988
	72	14.61 (6.77–31.53)	2	1.20 ± 0.17	0.980

a Degree of freedom

b Chi square

## 4 Discussion

The study was designed to evaluate the insecticidal efficacy of plant based oils and their admixed emulsion as a bio-pesticide to replace the harmful effects of synthetic insecticides from the environment [58]. Results confirmed the effectiveness of nano emulsions by controlling the population of *T. castaneum.* Toxicity effect was found increased with increase in concentration and exposure time. Essential oils of *C. citratus, T. ammi, O. basilicum,* and *C. longa* were found toxic to the adults of *T. castaneum*. Emulsion formation increased the toxicity of turmeric oil by 2% as mortality of *T. castaneum* was observed 100% after being exposed to turmeric emulsion as compared to turmeric oil (98%) at highest concentration. Reports of similar studies confirm the same toxicity pattern of phyto oils and their emulsions [26], [48], [55]*.* The increased insecticidal activity of emulsions is explained as increased insect cuticle penetration and lowering the α- amylases, and acetylcholine esterase in *T. castaneum* [59]. Similar results were obtained when turmeric nanoparticle was used on microbes [60]. The opposite phenomenon is observed when turmeric was tested on aphids (*Aphis gosypii*) [21]. Three per cent increase in the pesticidal efficacy of lemongrass oil was observed when its emulsion was used. Lemongrass oil resulted in up to 95% mortality in *T. castaneum* similar to *Sitophilus zeamasis* along with feeding repellency [2], [61]. Lemon grass emulsion exhibited 3% increased insecticidal activity (i.e., 98%) as compared to lemon grass oil which is a similar to nano application of *Hugona mystax* against mosquito [62]. Basil oil was comparatively less toxic to *T. castaneum* as compared to lemongrass oil and turmeric oil. The pesticidal activity of basil oil was enhanced by 5% when converted to emulsion. Significantly high adult mortality of 85% was noticed with basil oil. These results are quite similar to the study conducted on *Sitophilus oryzae* and *Culex pipiens* larvae [37], [63]. The well pronounced adulticidal and larvicidal effect of basil oil could be due to its neurotoxic activity of inhibiting acetylcholine esterase [37]. Basil oil is known to exhibit high repellence effects against *Aphis gosypii* and *Phenacoccus solenopsis* [64]. The mortality percentage increased to 90% when used as nano-emulsion against *T.castaneum* parallel to similar studies on *Aedes aegypti* [65]. Ajwain oil has a moderate effect on *T. castaneum* and resulted in 65% mortality. The efficacy increased in ajwain emulsion by 15%. The results of ajwain oil tested alone were equal to previously studied *Trogoderma granarium* and *Prostephanus truncatus* [66] and *Culex quinquefasciatus* [67]. Ajwain oil exhibit significant acetylcholine esterase enzyme inhibition due to thymol against larvae of *Tuta absoluta*. The comparatively low mortality in ajwain oil could be due to its relatively less effectiveness on adults [68]. Ajwain oil also exhibited significant results against *Fusarium oxysporum, Meloidogyne incognita,* and *Odontotermes obesus* [1]. Ajwain emulsion enhanced mortality by 68.33%. Similar effects were seen when ajwain microemulsion was used against *Culex quinquefasciatus* [69]*.* Diatomaceous earth was also used to test its effect on *T. castaneum* where it resulted 65% mortality. A slight contrast to these results is observed on *Sitophilus zeamais* where it caused 69% mortality [44]. The DE is reported effective in management of *Sitophilus oryzae*, *Tribolium confusum,* and *Rhyzopertha dominica* [70].

## 5 Conclusions

Plant based oils could serve as best possible economical alternative to synthetic pesticides usually employed for pest management. In the current study, essential oils from four plant species were evaluated against the adults of *T. castaneum.* Results from the study strongly support the use of turmeric, lemongrass, basil and ajwain as potential candidate in insecticidal formulations. Future need is to improve bioavailability of their major insecticidal compounds by developing them as micro and nano-carriers. The present study enlightens the fact that nano-formulations are more efficacious than oils and DE used alone. Development of plant oils as nano-emulsions could significantly enhance their penetration in insect cuticle. They can be utilized for sustainable management of storage pests on commercial grounds. The need of the hour is to further investigate phyto oils based nano emulsions for their mode of action, target site and safety towards non targets to develop new commercial formulation.

## CRediT authorship contribution statement

**Qamar Saeed, Syed Muhammad Zaka and Shaghef Ejaz** designed the experiment. **Maryam Tanveer, Tatheer e Zahra and Muhammad Saghir** performed the experiment. **Syed Muhammad Zaka** analysed the data. **Muazzama Batool** wrote the paper. **Qamar Saeed, Muazzama Batool and Syed Muhammad** zaka revised the paper.

## Declaration of Competing Interest

The authors declare that they have no known competing financial interests or personal relationships that could have appeared to influence the work reported in this paper.
